# *Garlic* Cellulosic Powders with Immobilized AgO and CuO Nanoparticles: Preparation, Characterization of the Nanocomposites, and Application to the Catalytic Degradation of Azo Dyes

**DOI:** 10.3390/polym16121661

**Published:** 2024-06-12

**Authors:** Nouha Sebeia, Mahjoub Jabli, Faridah Sonsudin

**Affiliations:** 1 Textile Materials and Processes Research Unit, Tunisia National Engineering School of Monastir, University of Monastir, Monastir 5000, Tunisia; 2 Department of Chemistry, College of Science, Majmaah University, Al-Majmaah 11952, Saudi Arabia; 3 Department of Chemistry, Faculty of Science, Universiti Malaya, Kuala Lumpur 50603, Malaysia

**Keywords:** garlic-CuO, garlic-AgO, catalytic degradation, naphthol blue black B, calmagite

## Abstract

Nanomaterials have attracted specific consideration due to their specific characteristics and uses in several promising fields. In the present study, *Chondrilla juncea* was employed as a biological extract to facilitate the reduction of copper and silver ions within garlic peel powders. The resulting garlic-CuO and garlic-AgO nanocomposites were characterized using several analytical methods including FTIR, TGA/DTG, SEM, TEM, and XRD analyses. The garlic peel exhibited a rough surface. The nanoparticles were evenly dispersed across its surface. The incorporation of CuO and AgO nanoparticles affected the crystal structure of garlic peel. The establishment of CuO and AgO nanoparticles was evidenced by the highest residual mass values observed for the prepared nanocomposites. The thermogravimetric analysis showed that the prepared nanocomposites had lower thermal stability compared with garlic peel powders. The prepared nanocomposites were used for catalytic degradation of naphthol blue black B and calmagite. The decolorization process depended on the quantity of H_2_O_2_, initial concentration of azo dyes, duration of contact, and temperature of the bath. The calculated activation energy (Ea) values for the garlic-CuO nanocomposites were found to be 18.44 kJ mol^−1^ and 23.28 kJ mol^−1^ for calmagite and naphthol solutions, respectively. However, those calculated for garlic-AgO nanocomposites were found to be 50.01 kJ mol^−1^ and 12.44 kJ mol^−1^ for calmagite and naphthol, respectively.

## 1. Introduction

Green preparation of nanomaterials, using natural products, constitutes an interesting topic due to the numerous advantages offered by this method compared with common chemical methods [1,2,3,4,5]. Indeed, natural products contain active molecules such as terpenoids, polyphenols, alkaloids, proteins, acids, etc. These biomolecules serve not only as reducing agents but also as capping agents for nanoparticles. Many natural substrates have been investigated in the literature for the purpose of designing nanostructures. These include capparious zeylanica [6], ocimum sanctum [7], vitis vinifera [8], azadirachta indica [9], symphyti radix [10], piper betle [11], phyllanthus plant [12], marine fungi [13], microorganisms [14], and many others.

The application of nanomaterials encompasses various fields such as sensors [15,16], medicine [17,18,19], water treatment [20,21,22], etc. In addition, significant attention has been directed toward the elimination of harmful contaminants from water through the implementation of nanostructured materials. The elimination of azo dyes, a type of pollutant found in water, has attracted high interest among researchers due to their harmful effects on aquatic and human life. Various methods, including oxidation [23], photocatalysis [24], electrochemical processes [25], and adsorption [26], have been studied to remove these hazardous contaminants from water. The utilization of H_2_O_2_ as an environmental oxidant confirms that oxidative degradation is indeed an effective approach for color removal. In addition, these approaches could employ biomaterials as carriers [27]. To our knowledge, several studies focused on the oxidative degradation of dyes using nanomaterials. For instance, Malakootian et al. [28] proposed a system constituted of phytochemical silver nanoparticles, H_2_O_2_, and ozone for reactive blue 19 degradation. Qing et al. [29] studied the potential of a AgO/H_2_O_2_ mixture to degrade ethyl violet and methylene blue. In our recent works, we investigated the oxidative degradation of some dye molecules using chitosan–copper oxide and pectin-MnO_2_ [30,31]. 

Therefore, it is necessary to find a suitable technique in order to efficiently treat and manage wastewater. It was demonstrated that filtration, biological treatments, oxidation, adsorption, flocculation, and membranes can be achieved by, importantly, using polymeric materials (including polymeric flocculants, polymeric filters, polymeric composites, polymeric membranes, etc.). Nano-adsorbents are generally considered more favorable for sustainable processing, including size controllable synthesis [32]. 

Garlic, renowned for its intriguing nature, has been utilized both as a culinary ingredient and for medicinal purposes. It is recognized as being valuable in tumor treatments, metabolic diseases, heart diseases, and it has anti-bacterial, and anti-inflammatory properties [33,34]. In the garlic cultivation sector, garlic straws and peels are well recognized as waste; simply, small amounts are used as food and most are cooked [35]. Chemically, garlic peel is constituted of cellulose, lignin, hemicellulose, and other extractives [36]. In the current research, garlic peel powders are used as carriers for the ecological preparation of copper and silver, and *Chondrilla juncea* as a biological extract. Several analytical techniques are used to analyze the resulting garlic-CuO and garlic-AgO nanocomposites, including FTIR, XRD, SEM, TEM, and TGA/DTG. Degradation experiments are assessed for naphthol blue black B and calmagite. The investigation focuses on examining the experimental factors that affect the decolorization process. These experimental factors include the quantity of H_2_O_2_, initial concentration of the dye, duration of contact, and temperature of the bath. To gain a deeper understanding of the decolorization mechanism involving calmagite and naphthol, garlic-CuO and garlic-AgO nanocomposites are utilized as catalysts, and 0th-order, 1st-order, and 2nd-order kinetic models are employed. Furthermore, free energy, entropy, activation energy, and enthalpy are determined by calculating the kinetic rate parameters at various temperatures.

## 2. Experimental

### 2.1. Materials and Reagents

**Figure 1:**
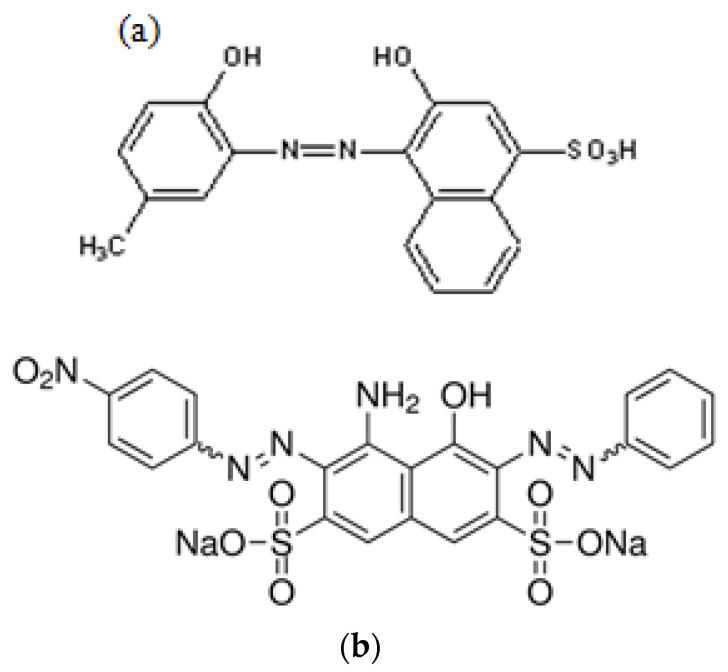
Chemical structures of (**a**) calmagite and (**b**) naphthol blue black B.

Garlic was bought from a local market (Zulfi, KSA). All chemicals and reagents were supplied by Sigma Aldrich and were of analytical grade. Naphthol blue black B (chemical formula: C_22_H_14_N_6_Na_2_O_9_S_2_, M.W = 616.5 g/mol, λ_max_ = 610 nm) and calmagite (chemical formula: C_17_H_14_N_2_O_5_S, M.W = 358.3 g/mol, λ_max_ = 538 nm) dyes were purchased to study catalytic degradation. The chemical structures of both dyes are displayed in Figure 1. H_2_SO_4_ and NaOH were used to adjust pH at constant values. Distilled water was used to prepare all aqueous solutions.

### 2.2. Preparation of Chondrilla juncea Aqueous Extract

**Figure 2:**
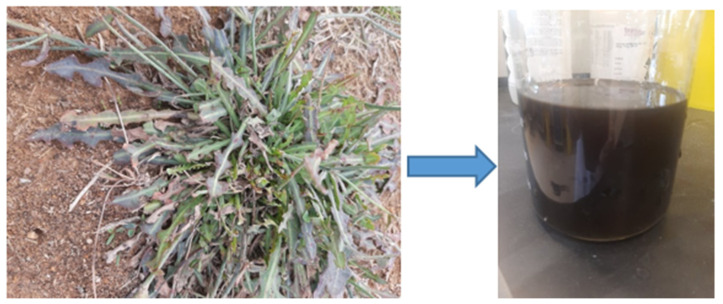
Preparation of extract from *Chondrilla juncea.*

*Chondrilla juncea* was collected from the region of Zulfi, Riyadh, KSA. The extraction protocol was the same as mentioned in our previous investigation [30]. The leaves were extracted manually and subsequently cleansed with distilled water to eliminate any impurities that adhered to their surface. Following this, they were dried in darkness for a period of 7 to 10 days. Once completely dried, the leaves were ground into a fine powder using an electric mixer. Next, a mixture containing 450 mL of water and 30 g of grinded leaves was prepared and heated for 120 min at 70 °C. Subsequently, the solution was allowed to cool down to ambient temperature and then filtered; the resulting extract (Figure 2) was utilized for the synthesis of garlic-CuO and garlic-AgO nanocomposites.

### 2.3. Synthesis of Garlic-CuO and Garlic-AgO Nanocomposites

The initial step involved grinding the dried garlic peel into a fine powder using an electric pro grinder device. The garlic straw powders were rinsed repeatedly with water to remove impurities and were dried at 50 °C overnight. Afterward, 5 g of garlic powder underwent treatment with solutions of copper sulfate and silver nitrate (1 M, volume = 250 mL) for 6 h. Next, after the addition of 250 mL of a freshly prepared *Chondrilla juncea* solution into pretreated garlic powder, an additional time of 2 h was allotted for the temperature to be raised to 70 °C. The gradual change of color solution throughout the reaction might indicate the conversion of copper and silver ions into nanoparticles. The resulting materials were separated by filtration, washed many times with distilled water, and dried at room temperature. Further, various analytical methods were employed to examine the structure of the resulting nanocomposites. These materials were subsequently utilized for the catalytic decomposition of naphthol blue black B and calmagite dyes.

### 2.4. Characterization Techniques

The studied materials were examined using a Perkin spectrum FTIR instrument (KSA). The spectra were displayed from a range of 400 to 4000 cm^−1^, with a resolution equal to 4 cm^−1^. A JEOL-5400 SEM (KSA) apparatus was used to evaluate the morphological characteristics. For this, compounds were covered with Au using a vacuum coater with the goal to enhance both image and conductivity qualities. The voltage of accelerating voltage was maintained at 20 kV. Thermal investigation was carried out in crucibles (Pt) in air and heated at a rate equal to 10°/min. Using a copper radiation source, the XRD-7000 SHIMADZU (KSA) (Kyoto, Japan) was employed to investigate X-ray diffraction patterns of the samples. The diffraction angle (2θ) was between 10° and 90°, the scanning speed was 2°/min, and the voltage and current were 40 kV and 40 mA, respectively. TEM images were obtained utilizing an 80 kV operated JEOL GEM-1010 transmission electron microscope (Tokyo, Japan).

### 2.5. Catalytic Degradation Experiments

Catalytic experimentations were performed in Erlenmeyer flasks enclosing a mixture of 20 mL of naphthol blue black B or calmagite and 0.01 g of garlic-CuO or garlic-AgO nanocomposites and stirred at 150 rpm. A measured dose of H_2_O_2_ was then added to the mixture. The pH of the solution was equal to 6, corresponding to the pH of the used water. When the experiment was supposed to reach equilibrium, the content was filtered with a filter paper. Then, absorbance of each colored solution was determined using a UV–Vis spectrophotometer at λ_max_ of 610 nm and 538 nm for naphthol blue black B or calmagite, respectively. The experiments were also carried out at different H_2_O_2_ doses (0–14 mL/L), contact times (0–10 min), temperatures (20–50 °C), and dye concentrations (10–50 mg/L).

## 3. Discussion of Results

### 3.1. FT-IR Exploration

**Figure 3:**
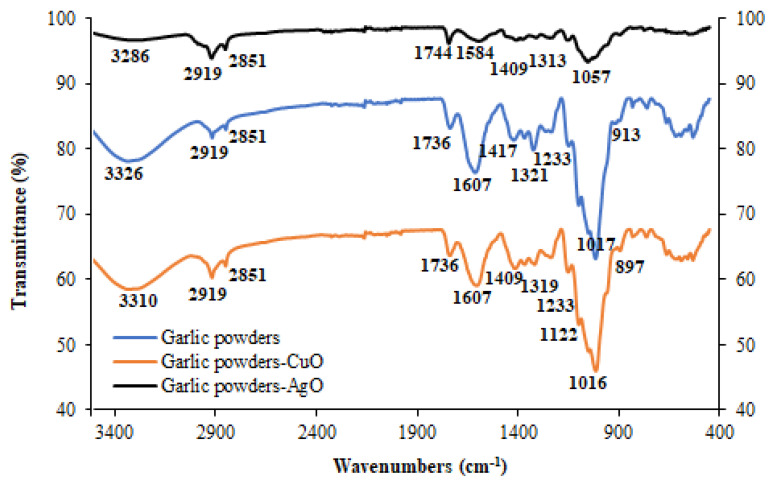
FT-IR of garlic powders, garlic powders-CuO, and garlic-AgO nanocomposites.

The FT-IR spectra of garlic peel and prepared garlic-CuO and garlic-AgO nanocomposites are displayed in Figure 3. The extensive peaks observed in the region of 3310 to 3286 cm^−1^ are the stretching vibration peaks of –OH [37]. The absorption peaks observed at 2919 and 2851 cm^−1^ are believed to be the stretching vibration peak of -CH in methyl and methylene [38]. The peak at 1744–1736 cm^−1^ is attributed to the C=O group present in hemicelluloses. The absorption peaks recorded at 1584–1607 cm^−1^ are attributed to the stretching vibration of C=C aromatic groups of lignin. The absorption peaks at 1417 cm^−1^ and 1321 cm^−1^ correspond to the bending vibration of -CH_2_ [39]. The peak of 1122 cm^−1^ is assigned to the asymmetric stretching vibration of C-O-C in cellulose. The absorption peak of 1017 cm^−1^ is attributed to the stretching vibration of the C-O group. The *β*-D-glucoside bond in the cellulosic material is observed at 913 cm^−1^. The comparison between the spectrum of the prepared garlic-CuO and garlic-AgO nanocomposites reveals that the main peaks of the studied materials remain similar, with a slight chemical shift during the chemical modification with silver nitrate and copper sulfate. For example, the position of the hydroxyl groups shifts from 3326 cm^−1^ to 3286 cm^−1^. Such a shift suggests that copper and silver ions interact with the garlic surface through the –OH groups. It is also worth noting that the biomolecules naturally found in the biological extract of *Chondrilla juncea* are able to reduce copper and silver ions into CuO and AgO nanoparticles. 

### 3.2. Morphological Investigation

**Figure 4:**
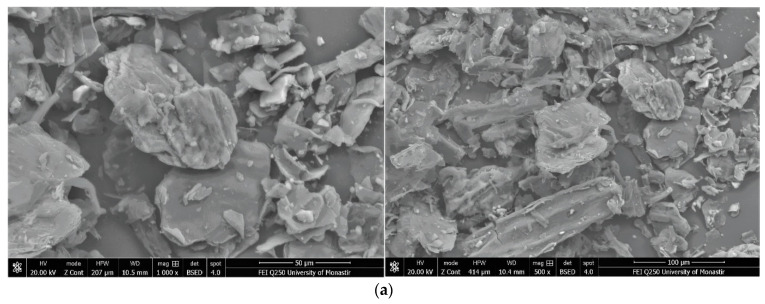

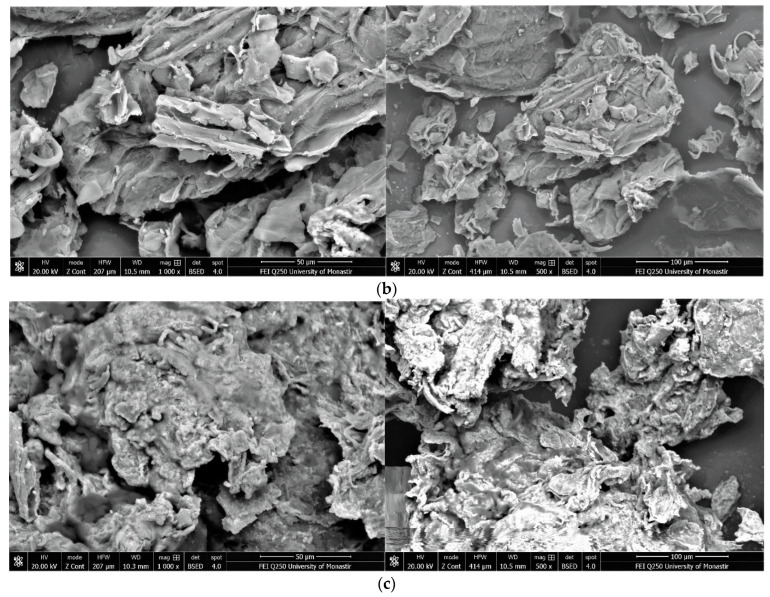
SEM images (magnifications ×500 and ×1000) from top to bottom: (**a**) garlic peel, (**b**) garlic-CuO, and (**c**) garlic-AgO nanocomposites.

**Figure 5:**
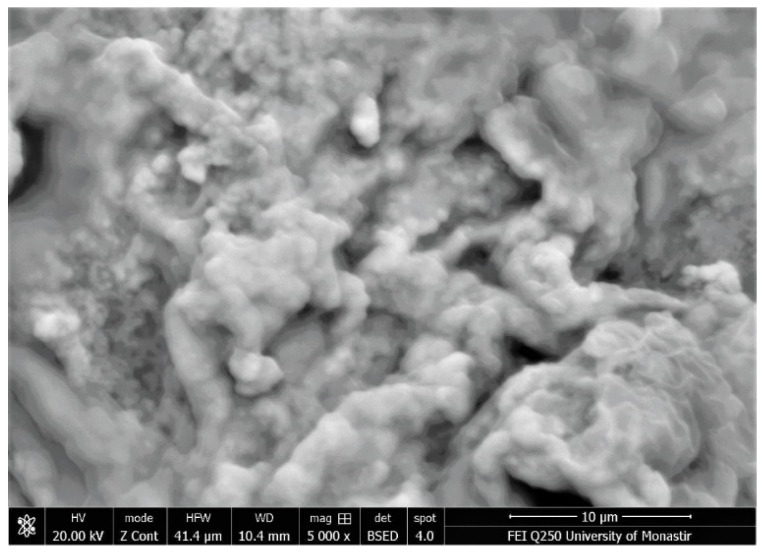
SEM images of garlic-AgO nanocomposites (magnification ×5000).

Figure 4 shows the SEM photos of garlic peel powders observed at different magnifications (×500 and ×1000) and the prepared garlic-CuO and garlic-AgO nanocomposites. As observed, the SEM images of the starting cellulosic material show particles of varied sizes and irregular shapes. After saturation with silver nitrate and copper sulfate ions and synthesis of CuO and AgO nanoparticles, the surfaces of the materials become rougher, and the particles are much more agglomerated, with greater swelling. Garlic-AgO nanocomposites show very significant material surface changes compared with garlic-CuO composites. Higher magnification (×5000) (Figure 5) of garlic-AgO images shows spherical AgO nanoparticles that are clearly observed and fully distributed on the surface of the garlic peel.

### 3.3. XRD Data

**Figure 6:**
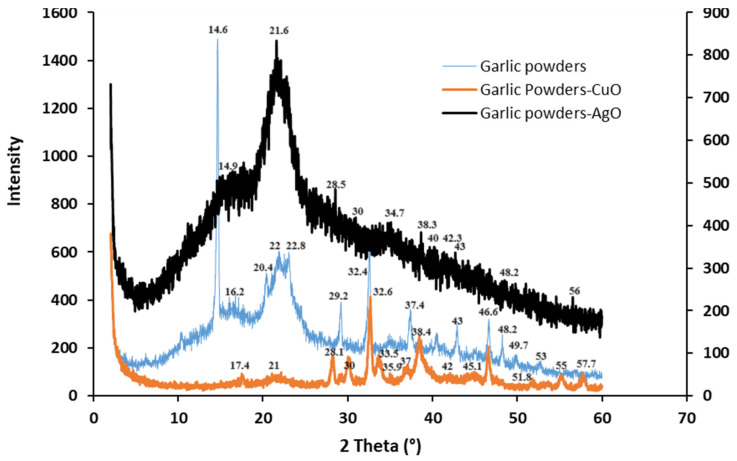
XRD patterns of garlic peel powders, garlic-CuO (JCPDS No: 98-009-2367), and garlic-AgO (JCPDS No. 74-1750) nanocomposites.

Figure 6 gives the XRD patterns of garlic peel powders, garlic-CuO, and garlic-AgO nanocomposites. As shown, the XRD data of the garlic peel powder display three diffraction peaks at 2θ = 16.2°, 22°, and 32.4° which correspond to the (101), (002), and (040) crystal planes, respectively. It is assigned to cellulose type I [40]. The strong diffraction peak, seen at 2θ = 29.2°, might be due to the presence of hemicellulose existing in cellulose [41]. Other observed diffraction hyphen might be assigned to the presence of other non-cellulosic constituents naturally existing in garlic peels. The three main diffraction peaks observed for garlic peel powders are also observed in the XRD pattern of the garlic-CuO nanocomposite but with slight shifting. They are observed at 2θ = 17.4°, 21°, and 32.6°. The peak intensities are significantly lower than that of garlic peel powders. The results show that, during the incorporation of CuO nanoparticles into garlic powders, the crystal structure of cellulose is changed. Moreover, the XRD pattern of the garlic-CuO nanocomposite exhibits the appearance of new diffraction peaks observed at 2θ = 33.5°, 35.9°, 37°, 38.4°, 42°, 45.1°, 46.6°, 51.8°, 55°, and 57.7°, which are related to (1 1 0), (1 1 −1), (1 1 1), (2 0 −2), (1 1 2), (2 0 2), (1 1 −3), (3 1 0), (1 1 3), and (2 2 1) planes of the CuO monoclinic phase (JCPDS No: 98-009-2367). The presence of other peaks in the range 2θ = 30°–38.4° proves the formation of CuO [42]. The XRD reflections of 2θ at 30°, 34.7°, 38.3°, 40°, 43°, 48.2°, and 56° can be ascribed to the (1−1-11), (002), (111), (2−2-02), (102), (2−2-12), and (1−1-13) crystallographic planes of monoclinic AgO (JCPDS No. 74-1750) [43].

### 3.4. TEM Images

**Figure 7:**
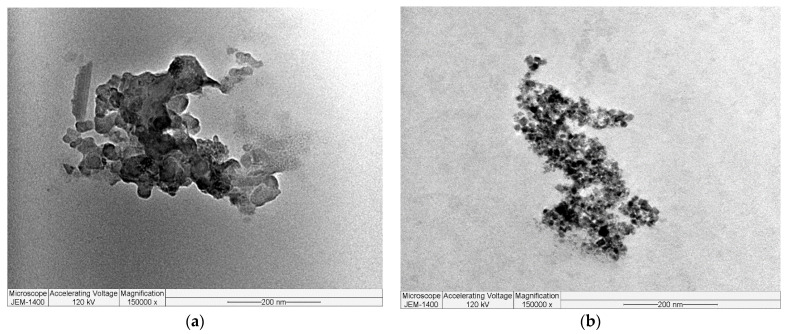
TEM pictures of (**a**) garlic-CuO and (**b**) garlic-AgO nanocomposites.

TEM pictures of garlic-CuO and garlic-AgO nanocomposites reveal a spherical morphology (Figure 7). The prepared nanoparticles generally demonstrate a spherical bent structure. The overlap of the synthesized nanoparticles results in diverse patterns justified by the presence of garlic peel powders as support to CuO and AgO nanoparticles. The TEM results also agree with FT-IR, XRD, and SEM results.

### 3.5. Thermal Examination

**Figure 8:**
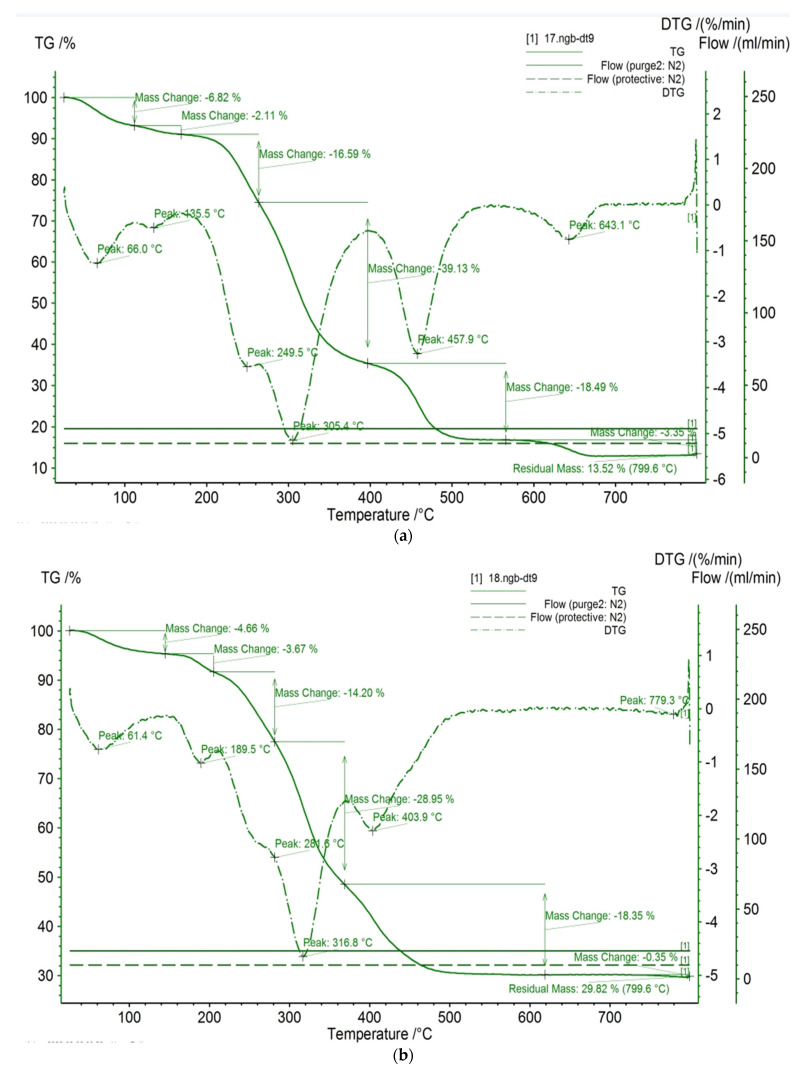

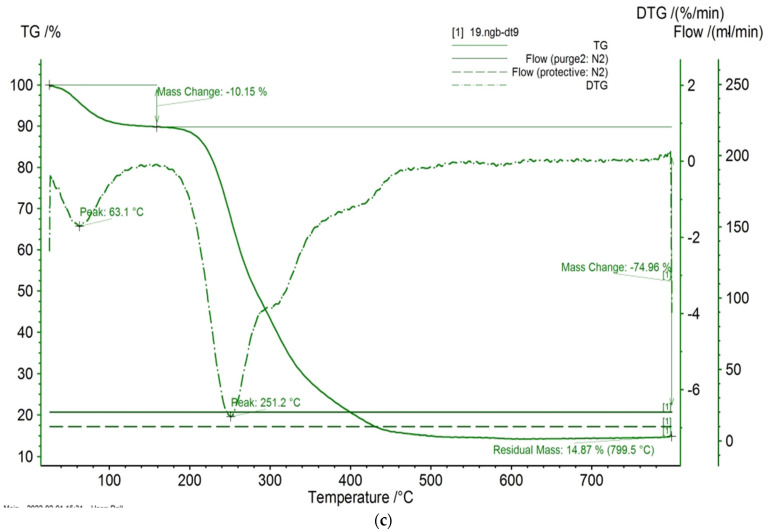
TGA/DTG of (**a**) garlic peel powders, (**b**) garlic-CuO, and (**c**) garlic-AgO nanocomposites.

TGA/DTG analysis is used to inspect the thermal strength of the studied compounds. Figure 8 depicts the TGA/DTG curves of garlic peel powders, garlic-CuO, and garlic-AgO nanocomposites. The thermal decomposition of garlic peel powders proceeds in different pyrolysis steps due to many reaction steps. As observed, at an initial stage, thermal curves show weight loss lower than 10% at 66 °C, 61.4 °C, and 63.1 °C for garlic powders, garlic-CuO, and garlic-AgO, respectively. This first weight loss is attributed to moisture evaporation and absorbed water in hydrophilic natural products [44]. The residual mass, achieved at 799.6 °C, is equal to 13.52 °C for garlic powders. The DTG curve of garlic powders reveals a series of thermal events observed at 136.5 °C, 249.5 °C, 305.4 °C, 457.9 °C, and 643.1 °C, which could be related to the essential pyrolytic reaction of cellulose and also the oxidation of the burned residues. The thermal events of garlic-CuO nanocomposites reveal that the residual mass, reached at 799.6 °C, is equal to 29.72 °C. The DTG curve of garlic-CuO nanocomposites shows a series of thermal decompositions observed at 189.5 °C, 281.6 °C, 316.8 °C, and 403.9 °C. However, the thermal events of garlic-AgO nanocomposites exhibit that the residual mass, reached at 799.6 °C, is equal to 14.87 °C. The DTA curve of garlic-AgO nanocomposites shows only one thermal decomposition observed at 251.2 °C. Indeed, the highest residual mass values observed for the prepared nanocomposites prove the formation of metal oxide nanoparticles. In addition, the thermal events prove that the prepared nanocomposites are thermally less stable than the garlic peel powders. Such a transformation in thermal results ensures again the chemical change in garlic powders. This change in thermal degradation after in situ synthesis of metal oxide nanoparticles indicates that the incorporation of CuO and AgO onto the surface of garlic affects the thermal behavior of the resulting materials. A different structure of garlic peel powder could be arranged. Possibly, the establishment of new bonds could faintly disturb the thermal stability of the studied material.

### 3.6. Degradation of Dye Solutions

#### 3.6.1. Effect of Experimental Conditions

**Figure 9:**
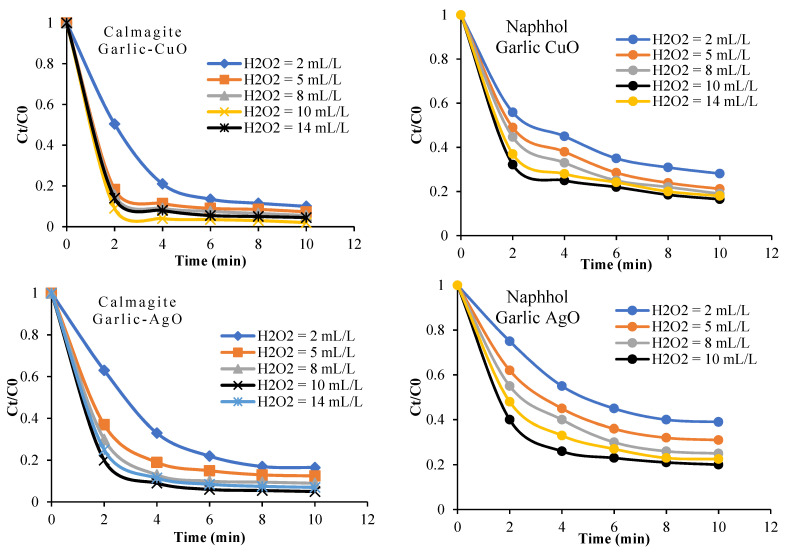
Effect of H_2_O_2_ on the catalytic degradation of calmagite and naphthol (T = 20 °C, pH = 6, C_0_ = 30 mg/L) solutions in the presence of garlic-CuO and garlic-AgO nanocomposites.

In this section, we discuss the influence of time of reaction, dye concentrations, and bath temperature on the degradation of naphthol blue black B and calmagite using the two binary garlic-CuO/H_2_O_2_ and garlic-AgO/H_2_O_2_ systems. Initially, we observe that naphthol and calmagite solutions are stable in the presence of H_2_O_2_ and no decolorization is shown. In addition, the adsorption of the two studied dyes onto nanocomposites alone is too small. However, after the addition of H_2_O_2_ into the dye solutions, the decolorization occurs in just a few minutes. Figure 9 gives the impact of the reaction time on the decolorization of naphthol and calmagite solutions. As observed, the decolorization yield of calmagite is equal to 98% in the garlic-CuO/H_2_O_2_ system after 10 min of reaction. However, the decolorization yield of naphthol is equal to 82% in the garlic-CuO/H_2_O_2_ system. This variation in the decolorization process between the two studied dyes is justified by the number of azo groups present in each dye structure and the difference in the molecular weight. Indeed, the highest yield of dye degradation is reached using the garlic-CuO nanocomposite. This might be related to the power of metal oxide itself. Overall, the highest degradation yields obtained within the prepared nanocomposites are explained by the combination of the radical OH• and garlic-CuO or garlic-AgO to break the azo groups, chromophoric groups of calmagite, and naphthol molecules. The maximum yield of decolorization is achieved using an amount of H_2_O_2_ equal to 10 mL/L. A high amount of H_2_O_2_ (14 mL/L) decreases the yield of decolorization. In fact, the accumulation of high amounts of oxidant into the solution undergoes self-quenching (scavenger) of OH• to yield HO_2_• radicals [45,46,47].

**Figure 10:**
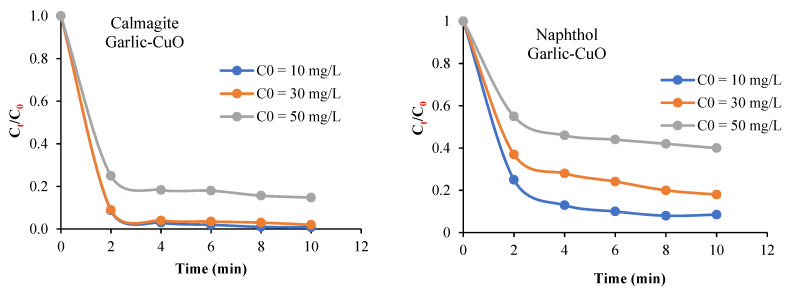

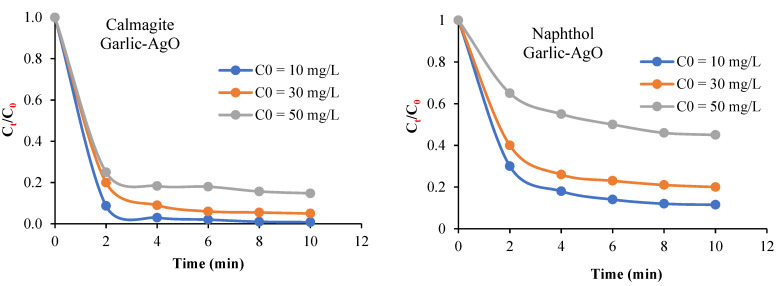
Effect of initial concentrations of calmagite and naphthol solutions (T = 20 °C, pH = 6, H_2_O_2_ = 10 mL/L) on catalytic degradation in the presence of garlic-CuO and garlic-AgO nanocomposites.

**Figure 11:**
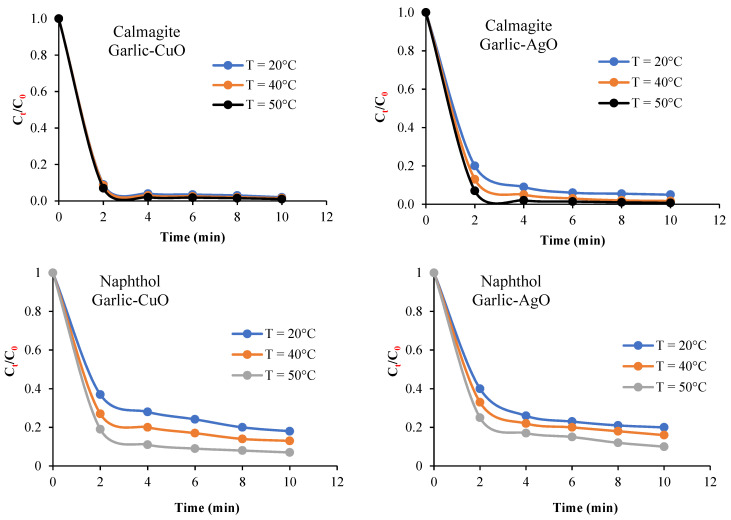
Effect of temperature on the catalytic degradation of calmagite and naphthol solutions (pH = 6, C_0_ = 30 mg/L, H_2_O_2_ = 10 mL/L) in the presence of garlic-CuO and garlic-AgO nanocomposites.

The efficiency of garlic-CuO and garlic-AgO nanocomposites/H_2_O_2_ to decolorize calmagite and naphthol solutions is also studied by varying the initial dye concentrations from 10 mg/L to 50 mg/L (Figure 10), while maintaining constant the amount of H_2_O_2_ at 8 mL/L. Results indicate that the decolorization yield decreases with the increase in initial dye concentrations. This indicates that there is only one optimum dose of H_2_O_2_ for every single initial dye concentration. As shown in Figure 11, the decolorization yield of the two studied solutions increases with the increase in bath temperature from 20 °C to 50 °C. This result proves that the degradation process is favored at high energies.

#### 3.6.2. Kinetic and Thermodynamic Investigations

In this study, 0th-order, 1st-order, and 2nd-order kinetic equations are used to better apprehend the decolorization process of calmagite and naphthol using garlic-CuO and garlic-AgO nanocomposites as catalysts. The expressions of the used kinetic equations are given in the following equations [48]:

(1) $$C_{t}=C_{0}-k_{0}t$$

(2) $$C_{t}=C_{0}e^{-k_{1}t}$$

(3) $$\frac{1}{C_{t}}=\frac{1}{C_{0}}+K_{2}t$$

where *C*_0_ is the initial dye concentration, *C_t_* is the concentration at time *t*, *k*_0–2_ are the apparent kinetic rate constants of 0th-, 1st- and 2nd-order kinetic equations, respectively.

**Figure 12:**
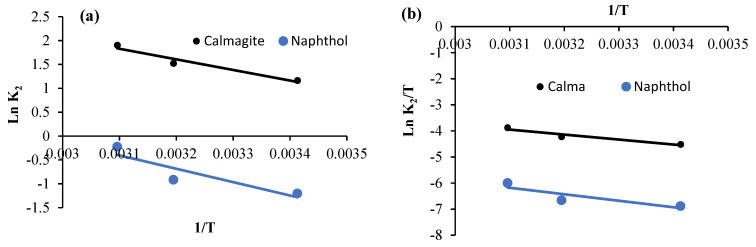
(**a**) Arrhenius plot and (**b**) plot of Ln (*K*_2_/*T*) vs. 1/*T* for calmagite and naphthol degradation using garlic-CuO nanocomposites.

**Figure 13:**
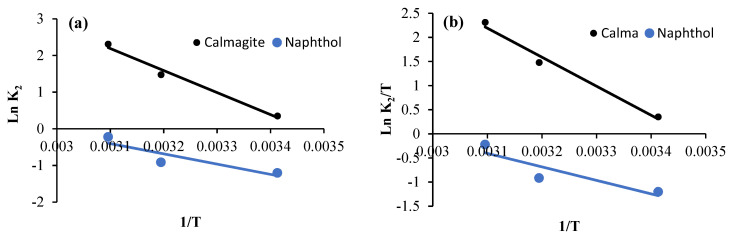
(**a**) Arrhenius plot and (**b**) plot of Ln (*K*_2_/*T*) vs. 1/*T* for calmagite and naphthol degrading using garlic-AgO nanocomposites.

When the values of *C_t_*, Ln *C_t_*/*C*_0_, and 1/*C_t_* are plotted vs. the time of reaction under the conducted conditions (H_2_O_2_ dose, temperature, and initial dye concentrations) (Figure 12 and Figure 13), the kinetic rate constants and regression coefficients are deduced. The values of the regression coefficients (*R*^2^) (Table 1 and Table 2) vary from 0.90 to 0.99 for the 2nd-order reaction kinetic, which are much higher than those determined for both 0th-order (0.47 < *R*^2^ < 0.78) and 1st-order (0.25 < *R*^2^ < 0.87) kinetic equations. Based on the obtained results, we can conclude that the decolorization of calmagite solution using garlic-CuO and garlic-AgO nanocomposites/H_2_O_2_ fits well to the 2nd-order reaction model.

The apparent rate constants, calculated at different temperatures, allow us to calculate entropy, activation energy, enthalpy, and free energy.

The values of the activation energies of the reaction, *E_a_* (kJ mol^−1^), are calculated according to the Arrhenius law [49]:

(4) $${K=Ae}^{-(\frac{E_{a}}{RT})}$$

where *A* is the Arrhenius factor, *T* is the temperature, *R* is the gas constant (J K^−1^ mol^−1^), and *K* is the calculated constant kinetic rate.

The values of ΔS* (J mol^−1^ K^−1^) and ΔH* (kJ mol^−1^) are determined using the Eyring formula: [50]:

(5) $$Ln\left(\frac{K}{T}\right)=Ln\;\left(\frac{K_{b}}{h}\right)+\frac{\Delta S{}^{\circ}}{R}-\frac{\Delta H{}^{\circ}}{RT}$$

where *K_b_* is the Boltzmann constant and *h* is the Plank constant. 

Ln K_2_ values are plotted vs. 1/*T* values. Then, gradients of the plots are used to define activation energy (Figure 12a and Figure 13a). However, activation enthalpy and entropy values are calculated using Ln (*K*_2_/*T*) plots vs. 1/*T* (Figure 12b and Figure 13b). Finally, activation energy (kJ mol^−1^) is calculated from Equation (6):

(6) $$\Delta G^{0}={\Delta H}^{0}-\;T.\Delta S^{0}$$

The negative values of entropy of the system indicate the fall of the disorder during dye degradation, while the positive values of the enthalpy prove that the studied system is endothermic, which is consistent with the increase in decolorization when the temperature increases.

*E_a_* values calculated for garlic-CuO nanocomposite are equal to18.44 kJ mol^−1^ and 23.28 kJ mol^−1^ for calmagite and naphthol solutions, respectively (Figure 12 and Figure 13). However, *E_a_* values calculated for garlic-AgO nanocomposite are equal to 50.01 kJ mol^−1^ and 12.44 kJ mol^−1^ for calmagite and naphthol solutions, respectively. The calculated low *E_a_* values suggest that the prepared nanocomposites are efficient for the degradation of calmagite and naphthol solutions. It is noteworthy to clarify that the calculated *E_a_* values are comparable to results reached using some other prepared compound-based catalysts for the degradation of different organic molecules investigated in the literature [23,31,51,52,53,54,55,56]. 

## 4. Conclusions

In summary, *Chondrilla juncea* was used as a biological extract to prepare stable CuO and AgO nanoparticles into garlic peel powders. The evidence of the formation of garlic-CuO and garlic-AgO nanocomposites was confirmed using FTIR, XRD, SEM, TEM, and TGA/DTG analyses. CuO and AgO nanoparticles were well distributed on the surface of the garlic peel. The incorporation of CuO and AgO nanoparticles affected the crystal structure of garlic peel. The thermal events confirmed that the prepared nanocomposites were thermally less stable compared with garlic peel powders, confirming again the incorporation of nanoparticles. The catalytic degradation experiments were assessed for naphthol blue black B and calmagite dye solutions. The decolorization mechanism depended on several experimental parameters such as amount of H_2_O_2_, initial dye concentration, time of contact, and bath temperature. The activation energy (Ea) values calculated for garlic-CuO nanocomposite were equal to 18.44 kJ mol^−1^ and 23.28 kJ mol^−1^ for calmagite and naphthol solutions, respectively. However, those calculated for garlic-AgO nanocomposite were found to be 50.01 kJ mol^−1^ and 12.44 kJ mol^−1^ for calmagite and naphthol, respectively. The calculated low Ea values suggested that the prepared nanocomposites could be used as efficient catalysts for the catalytic degradation of azo dye solutions. Further experiments will be extended for the preparation of new nanocomposite-based natural products for water remediation. Other toxic pollutants will be investigated including metals, phenolic compounds, pesticides, etc.

## Figures and Tables

**Table 1:** Kinetic rates and thermodynamic parameters calculated during degradation of calmagite using garlic-CuO nanocomposite/H_2_O_2._

Parameters	0th-Order		1st-Order		2nd-Order	
	*k*_0_	*R* ^2^	*k*_1_	*R* ^2^	*k*_2_	*R* ^2^
H_2_O_2_ amount (mL)						
2	0.11	0.75	0.27	0.87	0.69	0.97
5	0.099	0.52	0.33	0.50	0.86	0.95
8	0.10	0.52	0.36	0.53	1.17	0.96
10	0.101	0.50	0.47	0.52	3.20	0.96
Calmagite concentration (mg/L)						
10	0.104	0.50	0.58	0.74	9.20	0.96
30	0.101	0.50	0.47	0.52	3.20	0.96
50	0.09	0.53	0.25	0.35	0.37	0.90
Temperature (°C)						
20	0.101	0.50	0.47	0.52	3.20	0.96
40	0.102	0.51	0.52	0.56	4.58	0.98
50	0.103	0.47	0.56	0.55	6.70	0.96
Thermodynamic parameters						
Temperature (°C)	*E_a_* (kJ/mol)	ΔS* (J/mol/K)	ΔH* (kJ/mol)		ΔG* (kJ/mol)	
20	18.44	−0.18	15.89		68.96	
40					72.85	
50					74.40	

**Table 2:** Kinetic rates and thermodynamic parameters calculated during degradation of naphthol using garlic-CuO nanocomposite/H_2_O_2._

Parameters	0th-Order		1st-Order		2nd-Order	
	*k*_0_	R^2^	*k*_1_	R^2^	*k*_2_	R^2^
H_2_O_2_ amount (mL)						
2	0.102	0.78	0.15	0.83	0.16	0.98
5	0.109	0.75	0.18	0.83	0.29	0.99
8	0.09	0.71	0.20	0.78	0.23	0.99
10	0.093	0.64	0.21	0.65	0.30	0.96
Naphthol concentration (mg/L)						
10	0.012	0.74	0.58	0.74	9.17	0.97
30	0.093	0.64	0.21	0.65	0.30	0.96
50	0.104	0.53	0.25	0.35	0.40	0.90
Temperature (°C)						
20	0.093	0.64	0.21	0.65	0.30	0.96
40	0.109	0.57	0.25	0.54	0.40	0.94
50	0.114	0.53	0.33	0.52	0.80	0.95
Thermodynamic parameters						
Temperature (°C)	*E_a_* (kJ/mol)	ΔS* (J/mol/K)	ΔH* (kJ/mol)		ΔG* (kJ/mol)	
20	23.28	−0.19	20.74		74.84	
40					78.53	
50					80.38	

## Data Availability

The data presented in this study are available on request from the corresponding author.

## References

[1] Singh R., Wagh P., Wadhwani S., Gaidhani S., Kumbhar A., Bellare J., Chopade B.A. (2013). Synthesis, optimization, and characterization of silver nanoparticles from Acinetobacter calcoaceticus and their enhanced antibacterial activity when combined with antibiotics. Int. J. Nanomed..

[2] Shende S., Ingle A.P., Gade A., Rai M. (2015). Green synthesis of copper nanoparticles by Citrus medica Linn. (Idilimbu) juice and its antimicrobial activity. World J. Microbiol. Biotechnol..

[3] Zhang F., Chen X., Wu F., Ji Y. (2017). High adsorption capability and selectivity of ZnO nanoparticles for dye removal. Colloids and Surfaces A Physicochem. Eng. Asp..

[4] Lee Y., Choi J.R., Lee K.J., Stott N.E., Kim D. (2018). Large-scale synthesis of copper nanoparticles by chemically controlled reduction for applications of inkjet-printed electronics. Nanotechnology.

[5] Govindasamy R., Muthu T., Govindarasu M., Thandapani G., Chung I.-M. (2018). Green approach for synthesis of zinc oxide nanoparticles from Andrographis paniculata leaf extract and evaluation of their antioxidant, anti-diabetic, and anti-inflammatory activities. Bioproc. Biosys. Eng..

[6] Saranyaadevi K., Subha V., Ravindran R.S.E., Renganathan S. (2014). Synthesis and characterization of copper nanoparticle using Capparis Zeylanica leaf extract. Int. J. Chem. Tech. Res..

[7] Patel B.H., Channiwala M.Z., Chaudhari S.B., Mandot A.A. (2016). Biosynthesis of copper nanoparticles; its characterization and efficacy against human pathogenic bacterium. J. Environ. Chem. Eng..

[8] Angrasan J.K.V.M., Subbaiya R. (2014). Biosynthesis of copper nanoparticles by Vitis vinifera leaf aqueous extract and its antibacterial activity. Int. J. Curr. Microbiol. App. Sci..

[9] Nagar N., Devra V. (2018). Green synthesis and characterization of copper nanoparticles using Azadirachtaindica leaves. Mat. Chem. Phys..

[10] Aiste B., Viktorija J., Sandra S., Urte R., Jonas V., Patrick B.M., Pranas V. (2024). Antimicrobial Antioxidant Polymer Films with Green Silver Nanoparticles from Symphyti radix. Polymers.

[11] Ngoc-Thang N., Tien-Hieu V., Van-Huan B. (2023). Antibacterial and Antifungal Fabrication of Natural Lining Leather Using Bio-Synthesized Silver Nanoparticles from *Piper Betle* L. Leaf Extract. Polymers.

[12] Maxwell T., Nondumiso P.D., Douglas K., Amanda-Lee E., Manicum N.S.M.-F., Jacqueline V.T. (2023). Advances in Phytonanotechnology: A Plant-Mediated Green Synthesis of Metal Nanoparticles Using Phyllanthus Plant Extracts and Their Antimicrobial and Anticancer Applications. Nanomaterials.

[13] Basheer M.A., Khaled A., Nermine N.A.., Amal A.I.M. (2023). Mycosynthesis of silver nanoparticles using marine fungi and their antimicrobial activity against pathogenic microorganisms. J. Genet. Eng. Biotech..

[14] Namita A.S., Jagriti N., Deepa G., Vidhi J., Devendra P., Shariq S., Ravi K.S. (2023). Nanoparticles synthesis via microorganisms and their prospective applications in agriculture. Plant Nano Biol..

[15] Wang H., Rao H., Luo M., Xue X., Xue Z., Lu X. (2019). Noble metal nanoparticles growth-based colorimetric strategies: From monocolorimetric to multicolorimetric sensors. Coord. Chem. Rev..

[16] Amourizi F., Dashtian K., Ghaedi M. (2020). Polyvinylalcohol-citrate-stabilized gold nanoparticles supported congo red indicator as an optical sensor for selective colorimetric determination of Cr(III) ion. Polyhedron.

[17] Barrak H., Saied T., Chevallier P.L.G., M’nif A., Hamzaoui A.H. (2019). Synthesis, characterization, and functionalization of ZnO nanoparticles by N-(trimethoxysilylpropyl) ethylenediamine triacetic acid (TMSEDTA): Investigation of the interactions between Phloroglucinol and ZnO@TMSEDTA. Arab. J. Chem..

[18] Richard S., Boucher M., Lalatonne Y., Mériaux S., Motte L. (2017). Iron oxide nanoparticle surface decorated with cRGD peptides for magnetic resonance imaging of brain tumors. Biochim. Biophys. Acta (BBA) Gen. Subj..

[19] Safar R., Doumandji Z., Saidou T., Ferrari L., Nahle S., Rihn B.H., Joubert O. (2019). Cytotoxicity and global transcriptional responses induced by zinc oxide nanoparticles NM 110 in PMA-differentiated THP-1 cells. Toxicol. Lett..

[20] Bibi I., Nazar N., Iqbal M., Kamal S., Nawaz H., Nouren S., Safa Y., Jilani K., Sultan M., Ata S. (2017). Green and eco-friendly synthesis of cobalt-oxide nanoparticle: Characterization and photo-catalytic activity. Adv. Powder Technol..

[21] Issaabadi Z., Nasrollahzadeh M., Sajadi S.M. (2017). Green synthesis of the copper nanoparticles supported on bentonite and investigation of its catalytic activity. J. Clean. Prod..

[22] Reddy S.B., Mandal B.K. (2017). Facile green synthesis of zinc oxide nanoparticles by Eucalyptus globulus and their photocatalytic and antioxidant activity. Adv. Powder Technol..

[23] Hassani R., Jabli M., Kacem Y., Marrot J., Prim D., Hassine B.B. (2015). New Palladium-Oxazoline Complexes: Synthesis and Evaluation of the Optical Properties and the catalytic power during the oxidation of textile dyes. Beilst. J. Org. Chem..

[24] Hieu N.C., Lien T.M., Van T.T.T., Juan R.-S. (2020). Enhanced removal of various dyes from aqueous solutions by UV and simulated solar photocatalysis over TiO2/ZnO/rGO composites. Separat. Purif. Technol..

[25] Rosales E., Pazos M., Sanromán M.A. (2011). Comparative efficiencies of the decolourisation of leather dyes by enzymatic and electrochemical treatments. Desalination.

[26] Yagub M.T., Sen T.K., Afroze S., Ang H.M. (2014). Dye and its removal from aqueous solution by adsorption: A review. Adv. Colloid Interface Sci..

[27] Shen C., Song S., Zang L., Kang X., Wen Y., Liu W., Fu L. (2010). Efficient removal of dyes in water using chitosan microsphere supported cobalt (II) tetrasulfophthalocyanine with H_2_O_2_. J. Hazard. Mater..

[28] Malakootian M., Khatami M., Ahmadian M., Asadzadeh S.N. (2020). Biogenic silver nanoparticles/hydrogen peroxide/ozone: Efficient degradation of reactive blue 19. BioNanoScience.

[29] Qing W., Chen K., Wang Y., Liu X., Lu M. (2017). Green synthesis of silver nanoparticles by waste tea extract and degradation of organic dye in the absence and presence of H_2_O_2_. Appl. Surf. Sci..

[30] Syrine L., Mahjoub J., Saber B.A. (2021). Immobilization of copper oxide nanoparticles onto chitosan biopolymer: Application to the oxidative degradation of Naphthol blue black. Carbohy. Polym..

[31] Mahjoub J., Nouha S., Amor B. (2023). Synthesis and Characterization of Pectin-Manganese Oxide and Pectin-Tin Oxide Nanocomposites: Application to the Degradation of Calmagite in Water. J. Polym. Environ..

[32] Dimple P., Vinay S.B., Jyothi M.S., Ganesan S., Mahaveer K., Gurumurthy H. (2022). Garlic peel based mesoporous carbon nanospheres for an effective removal of malachite green dye from aqueous solutions: Detailed isotherms and kinetics. Spectrochimica Acta Part A Molecul. and Biomol. Spect..

[33] Arslaner A. (2020). The effects of adding garlic (*Allium sativum* L.) on the volatile composition and quality properties of yogurt. Food Sci. Technol..

[34] Liu Q., Fu Q., Du J., Liu X. (2022). Experimental study on the role and mechanism of allicin in ventricular remodeling through PPARα and PPARγ signaling pathways. Food Sci. Technol..

[35] Kallel F., Bettaieb F., Khiari R., García A., Bras J., Chaabouni S.E. (2016). Isolation and structural characterization of cellulose nanocrystals extracted from garlic straw residues. Ind. Prod..

[36] Jeevan P.R., Jong-Whan R. (2018). Extraction and Characterization of Cellulose Microfibers from Agricultural Wastes of Onion and Garlic. J. Nat. Fib..

[37] Nasef S., ElNesr E., Hafez F., Badawy N., Slim S. (2019). Gamma irradiation induced preparation of gum arabic/poly (vinyl alcohol) copolymer hydrogels for removal of heavy metal ions from wastewater. Arab. J. Nucl. Sci. Appl..

[38] Valentim R.M.B., Andrade S.M.C., Santos M.E.M., Santos A.C., Pereira V.S., Santos I.P., Dias C.G.B.T., Reis M.A.L. (2018). Composite based on biphasic calcium phosphate (HA/β-TCP) and nanocellulose from the açaí tegument. Materials.

[39] Hosseinzadeh S., Hosseinzadeh H., Pashaei S. (2019). Fabrication of nanocellulose loaded poly(AA-co-HEMA) hydrogels for ceftriaxone controlled delivery and crystal violet adsorption. Polym. Compos..

[40] Yan L., Wang L., Gao S., Liu C., Zhang Z., Ma A., Zheng L. (2020). Celery cellulose hydrogel as carriers for controlled release of short-chain fatty acid by ultrasound. Food Chem..

[41] Li X., Shu F., He C., Liu S., Leksawasdi N., Wang Q., Qi W., Alam M.A., Yuan Z., Gao Y. (2018). Preparation and investigation of highly selective solid acid catalysts with sodium lignosulfonate for hydrolysis of hemicellulose in corncob. RSC Adv..

[42] Nezamzadeh-Ejhieh A., Hushmandrad S. (2010). Solar photodecolorization of methylene blue by CuO/X zeolite as a heterogeneous catalyst. Appl. Catal. A Gen..

[43] Hua Y., Jing R.Z., Wentao C., Jin Z., Ji H.W. (2020). Screw-Dislocation-Driven Hierarchical Superstructures of Ag-Ag2O-AgO Nanoparticles. Crystals.

[44] Ramesh G.K., Subramanian S., Sathiyamurthy S., Prakash M. (2022). Calotropis gigantea fiber-epoxy composites: Influence of fiber orientation on mechanical properties and thermal behavior. J. Nat. Fibers.

[45] Aloui F., Jabli M., Hassine B.B. (2012). Synthesis and characterization of a new racemic helically chiral Ru (II) complex and its catalytic degradation of Eriochrome Blue Black B. Synth. Commun..

[46] Aloui F., Jabli M., Hassine B.B. (2013). New helically chiral metallated complexes: Characterization and catalytic activity. Synth. Commun..

[47] Banat S., Al-Asheh M., Al-Rawashdeh M., Nusair M. (2005). Photodegradation of methylene blue dye by the UV/H_2_O_2_ and UV/acetone oxidation processes. Desalination.

[48] Behnajady M.A., Modirshahla N., Ghanbary F. (2007). A kinetic model for the decolorization of C.I. Acid Yellow 23 by Fenton process. J. Hazard. Mater..

[49] Narayanan R.K., Devaki S.J., Rao T.P. (2014). Robust fibrillar nanocatalysts based on silver nanoparticle-entrapped polymeric hydrogels. Appl. Catal. A Gen..

[50] Sismanoglu T., Pura S. (2001). Adsorption of aqueous nitrophenols on clinoptilolite. Colloid Surf. A Physicochem. Eng. Asp..

[51] Jabli M., Touati R., Kacem Y., Hassine B.B. (2012). New chitosan microspheres supported [bis(2-methylallyl)(1,5-cyclooctadienne)ruthenium (II)] as efficient catalysts for colour degradation in the presence of hydrogen peroxide. J. Text. Inst..

[52] Guibal E., Vincent T. (2004). Chitosan-supported palladium catalyst. IV. Influence of temperature on nitrophenol degradation and thermodynamic parameters. J. Environ. Manag..

[53] Lea J., Adesina A.A. (2001). Oxidative degradation of 4-nitrophenol in UV-illuminated titania suspension. J. Chem. Technol. and Biotechnol..

[54] Jabli M., Baccouch W., Hamdaoui M., Aloui F. (2018). Preparation, characterization and evaluation of the catalytic power of an hybrid compound [H_3_PMo_12_O_40_-chitosan] for the azoic-colored solutions. J. Text. Inst..

[55] Zhang Q., Chuang K.T. (1998). Kinetics of wet oxidation of black liquor over a Pt–Pd–Ce/alumina catalyst. Appl. Catal. B Environ..

[56] Gallezot P., Laurain N., Isnard P. (1996). Catalytic wet-air oxidation of carboxylic acids on carbon-supported platinum catalysts. Appl. Catal. B Environ..

